# ICOS^+^PD-1^+^CXCR3^+^ T follicular helper cells contribute to the generation of high-avidity antibodies following influenza vaccination

**DOI:** 10.1038/srep26494

**Published:** 2016-05-27

**Authors:** Salah-Eddine Bentebibel, Surender Khurana, Nathalie Schmitt, Parvathi Kurup, Cynthia Mueller, Gerlinde Obermoser, A. Karolina Palucka, Randy A. Albrecht, Adolfo Garcia-Sastre, Hana Golding, Hideki Ueno

**Affiliations:** 1 Baylor Institute for Immunology Research, Baylor Research Institute, Dallas, TX, 75204, USA; 2 Division of Viral Products, Center for Biologics Evaluation and Research, U.S. Food and Drug Administration, Bethesda, MD 20892, USA; 3 Department of Microbiology, Icahn School of Medicine at Mount Sinai, New York, NY 10029, USA; 4 Global Health and Emerging Pathogens Institute, Icahn School of Medicine at Mount Sinai, New York, NY 10029, USA; 5 Department of Medicine, Division of Infectious Diseases, Icahn School of Medicine at Mount Sinai, New York, NY 10029, USA

## Abstract

The immune mechanism leading to the generation of protective antibody responses following influenza trivalent inactivated vaccine (TIV) vaccinations remains largely uncharacterized. We recently reported that TIV vaccination induced a transient increase of circulating ICOS^+^PD-1^+^CXCR3^+^ T follicular helper (cTfh) cells in blood, which positively correlated with the induction of protective antibody responses measured at day 28. However, whether and how these T cells directly contribute to antibody response remains unclear. In this study, we analyzed the changes after TIV vaccination in the amount and the avidity of the polyclonal antibodies specific for the HA1 subunit of the pandemic H1N1 virus, and analyzed the correlation with the increase of ICOS^+^PD-1^+^CXCR3^+^ cTfh cells. We found that both the amount and the avidity of specific antibodies rapidly increased during the first 7 days after TIV. Importantly, the increase of ICOS^+^PD-1^+^CXCR3^+^ cTfh cells strongly correlated with the increase in the avidity of antibodies, particularly in subjects who did not have high affinity antibodies at baseline. We propose that ICOS^+^PD-1^+^CXCR3^+^ Tfh cells directly contribute to the generation of high-avidity antibodies after TIV vaccinations by selectively interacting with high affinity B cells at extrafollicular sites.

Vaccination is the main strategy for prevention and control of seasonal influenza for the past 60 years^1^,^2^. Currently annual vaccination is recommended in the US with trivalent inactivated vaccine (TIV) for all individuals aged 6 months or older, or with live attenuated influenza vaccine (LAIV) for healthy non-pregnant people aged 2–49 years^3^. Nonetheless, a recent meta-analysis of clinical trials showed that the current influenza vaccine format provides protection only moderately. For example, 2009 pandemic H1N1 (pH1N1) vaccines were effective in only 60–93% (median 69%) of subjects younger than 65 years for prevention of influenza^2^. Although development of more effective influenza vaccines has long been desired, our current knowledge regarding the immune mechanism leading to the generation of protective antibody (Ab) responses following vaccinations is limited and insufficient for rational vaccine designs.

We recently reported that influenza TIV vaccinations transiently induced an emergence of a specific type of activated CD4^+^ helper T cells in blood^4^. These T cells expressed the chemokine receptor CXCR5 and co-stimulatory molecules ICOS and PD-1, and thus belong to a circulating compartment of T follicular helper cells (cTfh cells)^5^,^6^. Furthermore, the induced ICOS^+^PD-1^+^ cTfh cells expressed the chemokine receptor CXCR3, and displayed functional properties similar to Th1 cells including the production of IFN−γ. Importantly, the increase of ICOS^+^PD-1^+^CXCR3^+^ cTfh cells (which peaked at day 7 after TIV vaccination) positively correlated with the induction of protective antibody responses at day 28 (ref. 4). Furthermore, the induced ICOS^+^PD-1^+^CXCR3^+^ cTfh cells contained cells recognizing influenza antigens, and efficiently promoted influenza-specific memory B cells to differentiate into plasma cells *in vitro*^4^. These observations suggest the involvement of ICOS^+^PD-1^+^CXCR3^+^ cTfh cells in Ab responses, likely before they migrate out to the circulation. These observations were unexpected and perplexing, as multiple studies including ours demonstrated that CXCR3^+^ subset among cTfh cells was poor at providing help to B cells^7^,^8^,^9^. Furthermore, it is possible that the emergence of ICOS^+^PD-1^+^CXCR3^+^ cTfh cells reflects only a fraction of Tfh responses induced by TIV, and that other Tfh cells which remain in the lymph nodes are the major helpers directly associated with the Ab response. In this scenario, rather than the extrafollicular response in which B cells undergo minimal somatic hypermutations^10^, germinal centers (GCs) where *bona fide* Tfh cells select high affinity B cells that have undergone somatic hypermutations^11^ might be the major site of Ab response in TIV vaccination. Thus, whether and how ICOS^+^PD-1^+^CXCR3^+^ cTfh cells contribute to Ab response remains unclear.

In this study, we aimed at determining whether ICOS^+^PD-1^+^CXCR3^+^ Tfh cells emerging in blood were directly involved in the generation of Abs in TIV vaccination. Here we provide lines of evidence suggesting the direct contribution of ICOS^+^PD-1^+^CXCR3^+^ Tfh cells to the generation of high-avidity Abs.

## Results

### pH1N1 Ab maturation occurs within 7 days post TIV vaccination

Influenza vaccines provide protection mainly by generating high-avidity Abs against hemagglutinin^1^,^2^. Serum Ab titers against hemagglutinin are determined based on hemagglutination-inhibition (HI) and viral neutralization (VN). These titers are influenced by two parameters: the amount and the avidity. We first determined when the amount and the avidity of influenza specific Abs increase after TIV vaccination. Serum samples were obtained from 26 adult subjects at baseline and 7 days and 28 days after TIV vaccination in the year of 2011–12, the second year of the inclusion of the 2009 pandemic H1N1 (pH1N1) strain in the vaccine. The amount and the avidity of the polyclonal IgG specific for pH1N1 HA1 were analyzed using a real-time kinetics assay by surface plasmon resonance (SPR). For coating of the SPR chips, we used properly folded recombinant functional HA1 (amino acids 1–330; globular head) protein derived from A/California/07/2009 strain expressed in a bacterial system^12^.

**Figure 1:**
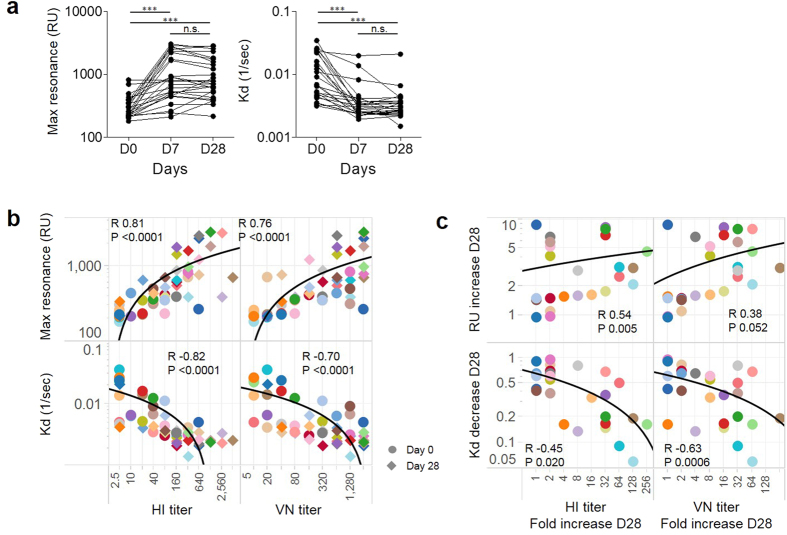
pH1N1 Ab maturation occurs within 7 days after TIV. (**a)** The amount (Max RU) and the avidity (Kd) of serum polyclonal Abs specific for pH1N1 HA1 at day 0, 7, and 28 post-TIV were determined by surface plasmon resonance. Paired t-test. ***p-value < 0.001, n = 26. (**b)** The correlation between Max RU and Kd of serum polyclonal Abs against pH1N1 HA1, and HI and VN titers. Results of day 0 and day 28 post-TIV. Spearman R and p-value are indicated. n = 52. (**c)** The correlation between the fold increase of Max RU/the fold decrease of Kd at day 28 and the increase of HI and VN titers at day 28. Spearman R and p-value are indicated. n = 26.

The binding of HA1-specific Ab (Max resonance unit (RU)), which reflects the amount of HA1-specific IgG^12^, significantly increased at day 7 compared to the baseline (1210 ± 190 RU *vs.* 320 ± 30 RU, Mean ± s.e.m., n = 26. p < 0.0001) (Fig. 1a). The steady-state off-rates (Kd) of HA1 antigen-Ab complexes, which reflects the avidity of HA1-specific IgG^12^, were significantly decreased at day 7 compared to the baseline (0.42 ± 0.08/sec *vs.* 1.22 ± 0.18/sec. p < 0.0001), indicating an increase in the avidity. Unexpectedly, neither the MaxRU nor the Kd did differ between day 7 and day 28. Thus, TIV vaccination increased the amount and the avidity of pH1N1 HA1-specific IgG within 7 days after vaccination, but not after day 7. Thus, the major antibody responses in TIV appears to complete within 7 days.

As expected, the Max RU of HA1-specific Abs in sera showed a positive correlation with the HI titers and VN titers (Spearman R 0.81 and 0.76, respectively, p < 0.0001 in both), while the Kd of HA1-specific Abs showed a negative correlation (Spearman R −0.82 and −0.70, respectively, p < 0.0001 in both) (Fig. 1b). We wondered which parameter in SPR contributed to the increase of Ab titers after the TIV vaccination. The decrease of Kd showed a negative correlation with the increase of both HI and VN titers (Spearman R −0.45 and −0.63, p-value 0.02 and 0.0006, respectively) (Fig. 1c). In contrast, while the increase of Max RU showed a positive correlation with the increase of HI titers (Spearman R 0.54, p = 0.005), it failed to show a correlation with the increase of VN titer (Spearman R 0.38, p > 0.05). Thus, while both increases in the amount and the avidity of specific Ab contribute to the increase of Ab titers in TIV vaccination, the increase in the avidity appears to dominantly contribute in particular to the increase of VN titer.

### Emergence of ICOS^+^PD-1^+^CXCR3^+^ cTfh cells correlates with an increase in the avidity

**Figure 2:**
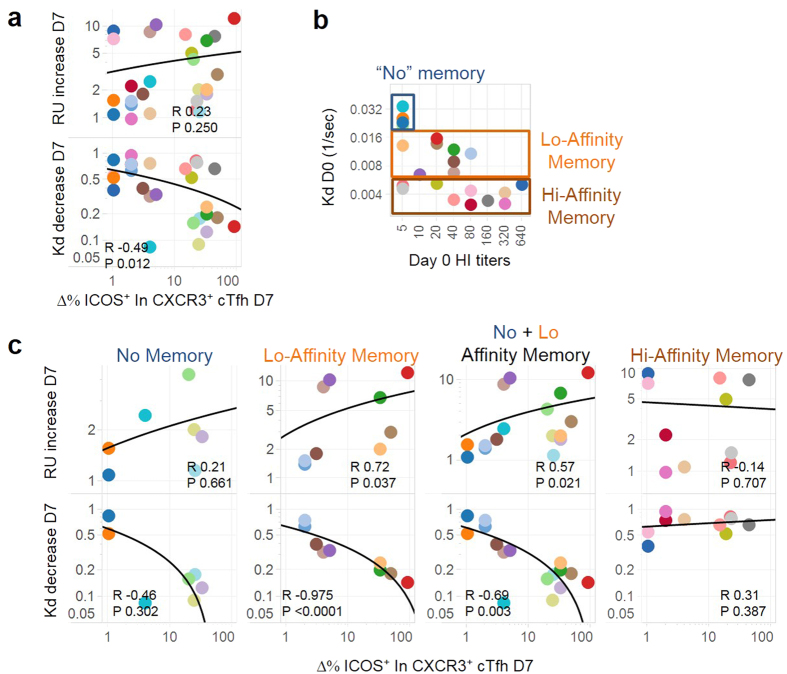
The increase of ICOS^+^PD-1^+^CXCR3^+^ cTfh cells correlates with the increase in Ab avidity. (**a)** The correlation between the increase of ICOS^+^PD-1^+^CXCR3^+^ cTfh cells at day 7 post-TIV and the fold increase of Max RU/the fold decrease of Kd at day 7. Spearman R and p-value are indicated. n = 26. (**b)** The three groups were defined in the subjects by using the results of Kd and HI titers at day 0. (**c)** The correlation between the increase of ICOS^+^PD-1^+^CXCR3^+^ cTfh cells at day 7 post-TIV and the fold increase of Max RU/the fold decrease of Kd at day 7 in each group.

We determined whether the increase of ICOS^+^PD-1^+^CXCR3^+^ cTfh cells at day 7 correlates with the increase in the amount and/or the avidity of specific Abs at day 7. We found that the increase of ICOS^+^PD-1^+^CXCR3^+^ cTfh cells correlated with the decrease in the Kd at day 7 (R = −0.49, p = 0.012), but not with the increase in the Max RU (R = 0.23, p = 0.25) (Fig. 2a). The same results were obtained with the SPR results measured at day 28 (not shown). This suggests that the emergence of ICOS^+^PD-1^+^CXCR3^+^ cTfh cells is associated with the increase in the avidity of specific Abs.

Yet the overall correlation between the increase of ICOS^+^PD-1^+^CXCR3^+^ cTfh cells and the decrease of the Kd was modest at best (R = −0.49, p = 0.012). We wondered whether the modest correlation might be due to the heterogeneity among the subjects in the quality and the quantity of pre-existing antigen-experienced B cells (which contain long-lived plasma cells and memory B cells). The integration of the results of Kd and HI titers at baseline led us separate the subjects into three subgroups: 1. “No memory” group, 2. “low affinity” memory group, and 3. “high affinity” memory group (Fig. 2b). “No memory” group was defined by HI titers less than 10 and high Kd (more than 0.02/sec), and thus had no to very few pre-existing antigen-experienced B cells capable of cross-recognizing the HA1 protein. “Low affinity” memory group was defined by medium Kd (between 0.006 and 0.02/sec), who had pre-existing antigen-experienced B cells that can cross-recognize HA1 only at low affinities. “High affinity” memory group was defined by low Kd (less than 0.006/sec), who had pre-existing antigen-experienced B cells that recognize HA1 at high affinities (Fig. 2b, Supplementary Figure 1).

We found that the percentage increase of ICOS^+^PD-1^+^CXCR3^+^ cTfh cells showed a very strong negative correlation with the decrease of the Kd in the “low affinity” memory group (R = −0.98, p < 0.0001, n = 9) (Fig. 2c). The increase of ICOS^+^PD-1^+^CXCR3^+^ cTfh cells in absolute number also correlated with the decrease of the Kd (R = −0.71, p = 0.03, Supplementary Figure 2). The integration of “no memory” and “low affinity” memory groups also showed a strong negative correlation (Fig. 2c, R = −0.69, p = 0.003, n = 16). In contrast, no correlation was observed in the “high-affinity” memory group, likely due to little increase in the avidity in this group. The increase of ICOS^+^PD-1^+^CXCR3^+^ cTfh cells also correlated with the increase of the MaxRU in “low affinity” memory group (R = 0.72, p = 0.04), and in the integration of the “no memory” and “low affinity” memory groups (R = 0.57, p = 0.02). However, in the “high-affinity” memory group, no correlation was observed between the increase of ICOS^+^PD-1^+^CXCR3^+^ cTfh cells and the increase of the MaxRU. Of note, when we classified the subjects according to the baseline HI titers (HI low ≤ 10, n = 12; HI mid between 20 and 40, n = 7; HI high ≥ 80, n = 7), the correlation between the increase of ICOS^+^PD-1^+^CXCR3^+^ cTfh cells and the decrease of the Kd or the increase of the MaxRU did not reach a statistical significance in any groups (Supplementary Figure 3a,b).

Collectively, the increase of ICOS^+^PD-1^+^CXCR3^+^ cTfh cells strongly correlated with the increase in the avidity and the amount of HA1-specific Abs in subjects who had few baseline high affinity antigen-experienced B cells. This suggests that the major role of ICOS^+^PD-1^+^CXCR3^+^ cTfh cells is to provide help exclusively to high affinity clones among the antigen-experienced B cell pool, and to promote their expansion. Because the frequency of high affinity clones is very low in these subjects, many ICOS^+^PD-1^+^CXCR3^+^ cTfh cells are likely required to expand high affinity clones and to increase the overall avidity (discussed later). In contrast, in subjects who had already high affinity antigen-experienced B cells at baseline, there was little increase in the Ab avidity. In these subjects, there is little pressure to select high affinity clones and probably only a small number of ICOS^+^PD-1^+^CXCR3^+^ cTfh cells are required to expand these clones.

### cTfh cells contain cells specific for multiple influenza antigens

We previously demonstrated that cTfh cells at day 7 post-TIV contained cells specific for influenza antigens^4^. For this analysis, PBMCs obtained at day 7 were stimulated with the TIV vaccine or heat inactivated influenza virus for 6 h in the presence of Brefeldin A and monensin, and the frequency of antigen-specific CD4^+^ T cells together with their cytokine expression pattern was determined by assessing CD4^+^ T cells that upregulated the expression of CD154 (ref. 4,13). In this assay, because the expression of CXCR5 and ICOS was not modified during the 6 h cell activation^4^, the frequency of antigen-specific cTfh cells could be characterized by assessing CD4^+^ T cells expressing CXCR5 and ICOS. Importantly, we demonstrated that influenza-specific cells were particularly enriched within ICOS^+^ cells among cTfh cells at day 7, the majority of which co-expressed CXCR3 (ref. 4). However, which influenza antigens these T cells recognized remains unclear.

**Figure 3:**
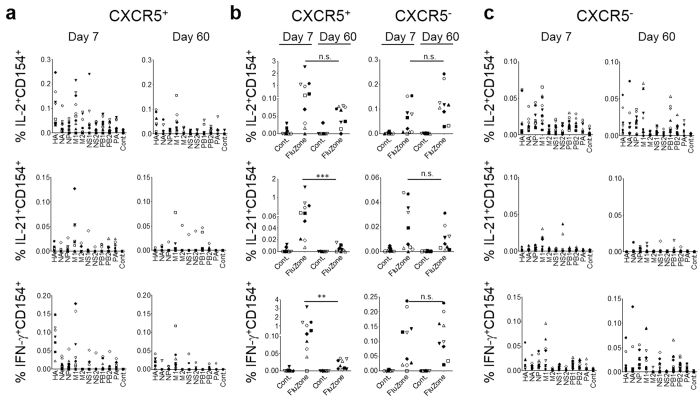
cTfh cells at day 7 post-TIV react to multiple influenza antigens. The CD154 assay was performed with PBMCs at day 7 and day 60 post-TIV and overlapping peptide libraries derived from hemagglutinin (HA), neuraminidase (NA), nucleoprotein (NP), matrix protein 1 and 2 (M1, M2), nonstructural protein 1 and 2 (NS1, NS2), polymerase proteins (PA, PB1, PB2) of the 2009 pandemic H1N1 strain. PBMC samples were obtained from at least 4 subjects and two assays were performed per sample on different days (indicated by closed and open symbols). The frequency of CD154^+^cytokine^+^ cells within CXCR5^+^CD4^+^ T cells and CXCR5^−^CD4^+^ T cells is indicated. (**a)** The CD154 assay was performed with PBMCs at day 7 and day 60 post-TIV and overlapping peptide libraries. The frequency of CD154^+^cytokine^+^ cells within CXCR5^+^CD4^+^ T cells is indicated. Mann-Whitney test. p-value ** < 0.01, *** < 0.001. (**b)** The results of the CD154 assay performed with PBMCs at day 7 and day 60 post-TIV and influenza vaccine (Fluzone^®^) or control (cont. PBS). The frequency of CD154^+^cytokine^+^ cells within CXCR5^+^CD4^+^ T cells and CXCR5^−^CD4^+^ T cells is indicated. (**c)** The results of the CD154 assay performed with PBMCs at day 7 and 60 post-TIV and overlapping peptide libraries. The frequency of CD154^+^cytokine^+^ cells within CXCR5^−^CD4^+^ T cells is indicated.

When interacting with T cells, B cells present peptides derived from antigens that they internalize via specific receptors. In case of TIV vaccination, B cells present peptides derived from any influenza proteins. Therefore, we hypothesized that the cTfh cells at day 7 post-TIV might be composed of a broad repertoire that recognize different influenza proteins. To address this, we analyzed the specificity of cTfh cells by using individual overlapping peptide libraries derived from each influenza protein including hemagglutinin (HA), neuraminidase (NA), nucleoprotein (NP), matrix protein 1 and 2 (M1, M2), nonstructural protein 1 and 2 (NS1, NS2), polymerase proteins (PA, PB1, PB2) of the 2009 pandemic H1N1 strain. The same approach probing the expression of CD154 (ref. 4) was used for the analysis. We found that cTfh cells at day 7 post-TIV were able to respond to each influenza proteins, and that the specific cTfh cells expressed IL-2, IL-21, and IFN-γ at various levels (Fig. 3a, Supplementary Figure 4). Among the influenza proteins, the response to HA and M1 was the most prevalent. These results show that cTfh cells at day 7 post-TIV were composed of a broad repertoire recognizing various influenza proteins.

We wondered whether influenza-specific cTfh cells were still detectable at a memory phase (day 60 post-TIV). In the CD154 assay with the stimulation of Fluzone^®^, influenza-reactive cTfh cells were still detectable at day 60, yet much reduced compared to day 7 (Fig. 3b). The frequency of cTfh cells specific for individual influenza proteins was also substantially decreased on day 60, yet those expressing IL-2 and/or IFN-γ in response to HA and M1 were still detectable (Fig. 3a). Of note, although blood CXCR5^−^CD4^+^ T cells (which contained both memory and naïve T cells in this assay) also contained influenza-reactive cells, (Fig. 3c, Supplementary Figure 5) the frequency and the cytokine production profiles were remarkably similar between day 7 and day 60 (Fig. 3b,c).

These results show that influenza-specific response is dynamic within cTfh cells following TIV vaccination, and the induced cTfh cells are composed of a broad repertoire. cTfh cells specific for multiple influenza proteins were still detectable at day 60 post-vaccination, suggesting their maintenance as memory cells.

## Discussion

In this study, we aimed at gaining insights into the role of ICOS^+^PD-1^+^CXCR3^+^ cTfh cells *in vivo* after TIV vaccination. Our study suggests that ICOS^+^PD-1^+^CXCR3^+^ Tfh cells directly contribute to the major antibody responses.

We found that both the amount and the avidity of specific Abs increased within 7 d after TIV vaccination. There was no further increase in the amount or the avidity after day 7. A previous kinetics study also showed that HI titers peak at day 10–14 and then plateau until day 30 (ref. 14). These observations suggest that the selection process of high affinity B cell clones initiates immediately after influenza vaccination, and almost completes within 7 days. While GCs may be formed after TIV vaccination, such rapid kinetics suggests that extrafollicular response is the major mechanism for the selection of high affinity clones following TIV vaccination.

**Figure 4:**
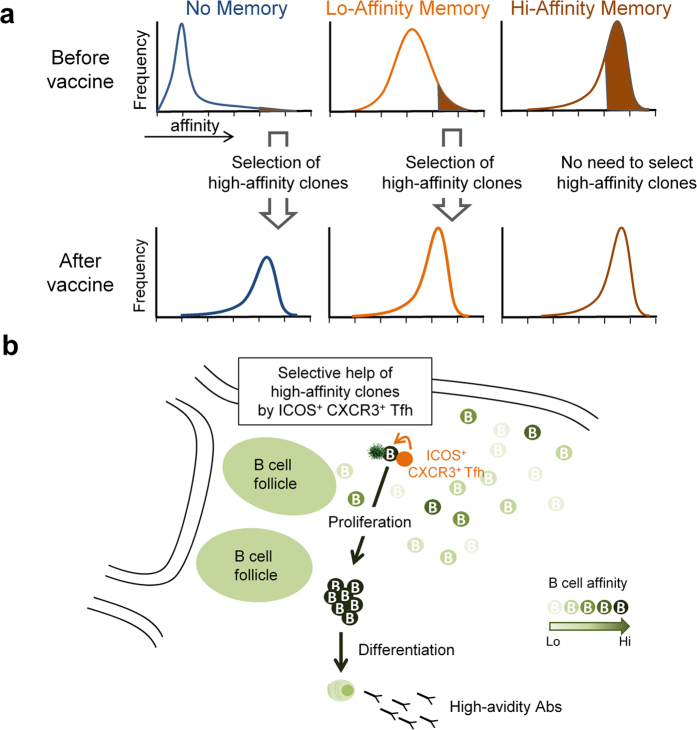
A model for the generation of high-avidity Abs in TIV vaccination. (**a)** Healthy individuals have a broad range of pre-existing antigen-experienced B cells that recognize a specific HA1 epitope at different affinities. When vaccination successfully induces a preferential expansion of high affinity clones and their differentiation into Ab-producing cells, vaccination induces an increase in the avidity of Abs. (**b)** High affinity B cells have an advantage over low affinity cells to capture and present antigens to ICOS^+^PD-1^+^CXCR3^+^ Tfh cells for cognate interactions. These interactions occur at extrafollicular sites, and induce high affinity clones to undergo extensive proliferation and differentiation into Ab-producing cells. ICOS^+^PD-1^+^CXCR3^+^ Tfh cells are composed of heterogeneous populations that recognize wide range of influenza proteins, which contributes to increase the chance to engage cognate interactions with any high affinity clones. Such selective activation and expansion of high affinity B cells contribute to the rapid increase of Ab avidity.

Then, how were high affinity clones selected? There is compelling evidence that antigen-experienced B cells but not naïve B cells are the major precursors of Ab-producing cells after influenza vaccinations^15^,^16^,^17^,^18^. Due to yearly vaccinations and occasional natural infections, healthy adults display a wide repertoire of antigen-experienced B cells that cross-recognize hemagglutinin of various influenza strains at different affinities^19^. Therefore, the success of TIV vaccination is likely dependent on the expansion of the right repertoire of antigen-experienced B cells, and an increase of Ab avidity can be achieved by preferentially expanding high affinity clones to HA of a specific virus strain (Fig. 4a). We observed only a marginal increase in the avidities after TIV vaccination in subjects who already had high-avidity Abs at baseline, suggesting that further selection of existing high affinity clones provides little impact in these subjects. Indeed, it is well known that subjects with high baseline titers tend to poorly respond to TIV vaccines^20^. Probably these subjects require only a few ICOS^+^PD-1^+^CXCR3^+^ cTfh cells to expand pre-existing high affinity B cell clones. On the other hand, in subjects who did not display high-avidity Abs (thus who would gain the largest benefit from TIV vaccinations. The majority of pre-existing B cells cross-react to the antigen null or only weakly), the increase of ICOS^+^PD-1^+^CXCR3^+^ cTfh cells strongly correlated with the increase in the avidity of Abs. This suggests that the selection process of pre-existing high affinity clones in these subjects is more demanding than in “high memory” group, and a larger number of ICOS^+^PD-1^+^CXCR3^+^ cTfh cells are required to expand high affinity clones and to increase the overall Ab avidities. As proposed for the selection process in GCs^21^, high affinity B cell clones probably have an advantage over low affinity clones in capturing and presenting antigens to T cells, and the induced ICOS^+^PD-1^+^CXCR3^+^ cTfh cells preferentially interact with high affinity clones and induce their proliferation and differentiation. As shown in this study, cTfh cells induced after TIV vaccination were composed of a broad repertoire recognizing different influenza antigens, and thus are capable of interacting with a wide array of high affinity B cell clones.

Of note, we did not see a clear correlation between the increase of ICOS^+^PD-1^+^CXCR3^+^ cTfh cells and the increase in the avidity of Abs in “no memory” group. We previously showed that ICOS^+^PD-1^+^CXCR3^+^ cTfh cells displayed an ability to induce memory B cells to become antibody-producing cells, but lacked the ability to induce naïve B cells to do so^4^. We also showed that an increase of ICOS^+^PD-1^+^CXCR3^+^ cTfh cells poorly correlated with an increase of pH1N1 HI titers in children who had never been exposed to pH1N1 (Ref. 4). Thus, some donors in the “no memory” group might have been “totally naïve” to pH1N1, and might have had few cross reactive antigen-experienced B cells at baseline.

As discussed earlier, the site that ICOS^+^PD-1^+^CXCR3^+^ cTfh cells interact with high affinity B cells seems to be extrafollicular area. This is also supported by other evidence. First, it was demonstrated that high affinity B cells preferentially contribute to extrafollicular response, while low-affinity B cells are preferentially directed to GCs where they undergo affinity maturation^22^. Second, ICOS^+^ cTfh cells emerging in blood after vaccination were shown to be derived from Tfh precursors rather than GC Tfh cells in mice models^23^,^24^. Collectively, we propose that ICOS^+^PD-1^+^CXCR3^+^ Tfh cells induced by TIV interact with antigen-experienced B cells at extrafollicular sites and contribute to the selection of high affinity clones (Fig. 4b). This model also explains well why the kinetics of the appearance of ICOS^+^PD-1^+^CXCR3^+^ cTfh cells in blood is identical with that of Ab-producing B cells after TIV vaccination^4^,^23^.

A majority of the induced ICOS^+^PD-1^+^CXCR3^+^ Tfh cells appeared to be short-lived, because the frequency of influenza-specific cTfh cells was substantially lower at day 60 than at day 7. Consistently, the frequency of ICOS^+^ cTfh cells peaked at day 7 post vaccination and returned to the baseline before day 28 (ref. 4,23). Nonetheless, we found that influenza-specific cTfh cells (in particular those specific for HA and M1) were still detectable at day 60 post vaccination. This suggests that a fraction of the induced specific cTfh cells remain as memory cells. Although how long these memory cTfh cells remain in the circulation and whether these cells contribute to Ab response upon next challenge remain to be established, this observation further support the importance of the generation of ICOS^+^PD-1^+^CXCR3^+^ Tfh cells after TIV vaccination.

In summary, our study strongly suggests that ICOS^+^PD-1^+^CXCR3^+^ Tfh cells directly contribute to the generation of Ab response in TIV vaccination. Generation of ICOS^+^PD-1^+^CXCR3^+^ Tfh cells is important for the generation of protective antibody response in influenza vaccine, in particular for subjects who have few highly cross-reactive antigen-experienced B cells. Accordingly, we surmise that ICOS^+^PD-1^+^CXCR3^+^ Tfh cells are also likely critical for the selection and the expansion of clones producing broadly neutralizing antibodies^25^. Our study will provide an important implication for rationale influenza vaccine designs.

## Methods

### Clinical samples

Baylor Health Care System review boards approved this study. The methods were carried out in accordance with the approved guidelines, and all experimental protocols were approved by Baylor Health Care System review boards. Blood samples were obtained from 26 healthy control subjects before and after the administration of a single intramuscular dose of a non-adjuvanted trivalent split seasonal influenza vaccine (2011/2012 Fluzone^®^, Sanofi Pasteur), the second year of the inclusion of pandemic H1N1 strain in the vaccine^4^. Informed consent was obtained from all subjects.

### Flow cytometry

Whole blood samples were used for phenotypic analysis of CD4^+^ T cells by flow cytometry as previously described^4^. Briefly, whole blood samples (200 μL) were incubated with the indicated antibodies and LIVE/DEAD fixable Aqua for 15 minutes at room temperature. The following antibodies were used: CXCR5 (1G10), CD3 (UCHT1), CD8 (SK1), CD4 (RPA-T4), CCR6 (11A9), CXCR3 (1C6/CXCR3), ICOS (C398.4A), CD45RA (2H4), and CD45 (HI30). The gating strategy of ICOS^+^PD-1^+^CXCR3^+^ cTfh cells was described previously^4^,^26^. Briefly, total CD4^+^ T cells were first gated as CD3^+^CD4^+^CD8^−^LIVE/DEAD^−^ cells, and then cTfh cells were gated as a CD45RA^−^CXCR5^+^ cell population. Among cTfh cells, CXCR3^+^CCR6^−^ cells were defined in this study as CXCR3^+^ cTfh cells, and the percentage of ICOS^+^ cells among CXCR3^+^ cTfh cells was analyzed. ICOS^+^ CXCR3^+^ cTfh cells were all expressed high levels of PD-1 (ref. 4).

### Antibody assays

Hemagglutination inhibition (HI) and virus neutralization (VN) antibody titers for each strain of influenza virus incorporated in the vaccines were determined at baseline and at indicated days post-vaccination as previously described^4^.

### Surface Plasmon Resonance

The amount and the avidity of the polyclonal IgG specific for pandemic H1N1 HA1 were determined directly from human sera tested in serial dilution before and after vaccination with Fluzone using a real-time kinetics assay by surface plasmon resonance (SPR) as previously described^12^. Briefly, Kinetic interactions of the polyclonal IgG with recombinant HA1 (rHA1) protein were monitored at 25 °C using a ProteOn SPR biosensor (BioRad). In the current study, we have assessed two parameters: 1) The average binding of HA1-specific Ab (Max resonance unit (RU)), which reflects the amount of HA1-specific IgG and 2) The antibody off-rate (Kd), a constant which describe the stability of the antigen-antibody complex that decays per second. The antibody off-rate (Kd) was determined from the serum sample interaction with recombinant HA1 (amino acids 1 to 330 globular head) protein derived from A/California/07/2009 strain using SPR in the dissociation phase and calculated using ProteOn manager software (BioRad).

### CD154 assay

The detailed method was described previously^4^. Briefly, PBMCs at day 7 post-vaccination were stimulated for 6 h with Fluzone^®^, or a pooled overlapping peptide library derived from each influenza protein, including hemagglutinin (HA), neuraminidase (NA), nucleoprotein (NP), matrix protein 1 and 2 (M1, M2), nonstructural protein 1 and 2 (NS1, NS2), polymerase proteins (PA, PB1, PB2) of A/California/07/2009 strain (all obtained from BEI resources), in the presence of Brefeldin A and monensin for the last 4 h, and expression of intracytoplasmic cytokines together with CD154 was analyzed. After the stimulation period, cells were subsequently incubated with anti-CD3 (UCHT1), anti-CD4 (S3.5), anti-CXCR5 (RF8B2), and Aqua live/Dead (Invitrogen). After permeabilization, cells were stained intracellularly with the following combination of antibodies: anti-IL-2 (MQ1-17H12), anti-IL-21 (3A3-N2.1), anti-IFN-γ (4S.B3), and CD154 (24–31) for 30 min at room temperature. The stained cells were acquired with LSR Fortessa.

## Additional Information

**How to cite this article**: Bentebibel, S.-E. *et al*. ICOS^+^PD-1^+^CXCR3^+^ T follicular helper cells contribute to the generation of high-avidity antibodies following influenza vaccination. *Sci. Rep.* **6**, 26494; doi: 10.1038/srep26494 (2016).

## Supplementary Material

Supplementary Information
