# Supplementary material for: ICOS+PD-1+CXCR3+ T follicular helper cells contribute to the generation of high-avidity antibodies following influenza vaccination
Source: Sci Rep. 2016 May 27;6:26494. doi: 10.1038/srep26494 (PMC4882544; doi:10.1038/srep26494)
Supplement: Supplementary Information [file srep26494-s1.pdf]

**Supplementary Figures for**

# ICOS$^+$PD-1$^+$CXCR3$^+$ T Follicular Helper Cells Contribute to the Generation of High Avidity Antibodies Following Influenza Vaccination

Salah-Eddine Bentebibel[1]*, Surender Khurana[2]*, Nathalie Schmitt[1], Parvathi Kurup[1], Cynthia Mueller[1], Gerlinde Obermoser[1], A. Karolina Palucka[1], Randy A. Albrecht[3,4], Adolfo Garcia-Sastre[3,4,5], Hana Golding[2], and Hideki Ueno[1,6]

[1]Baylor Institute for Immunology Research, Baylor Research Institute, Dallas, TX, 75204, USA

[2]Division of Viral Products, Center for Biologics Evaluation and Research, U.S. Food and Drug Administration, Bethesda, MD 20892, USA.

[3]Department of Microbiology, [4]Global Health and Emerging Pathogens Institute, [5]Department of Medicine, Division of Infectious Diseases, Icahn School of Medicine at Mount Sinai, New York, NY 10029, USA

[6]Current address: Department of Microbiology, Global Health and Emerging Pathogens Institute, Icahn School of Medicine at Mount Sinai, New York, NY 10029, USA

**Figure S1: Ab response in the three subgroups**

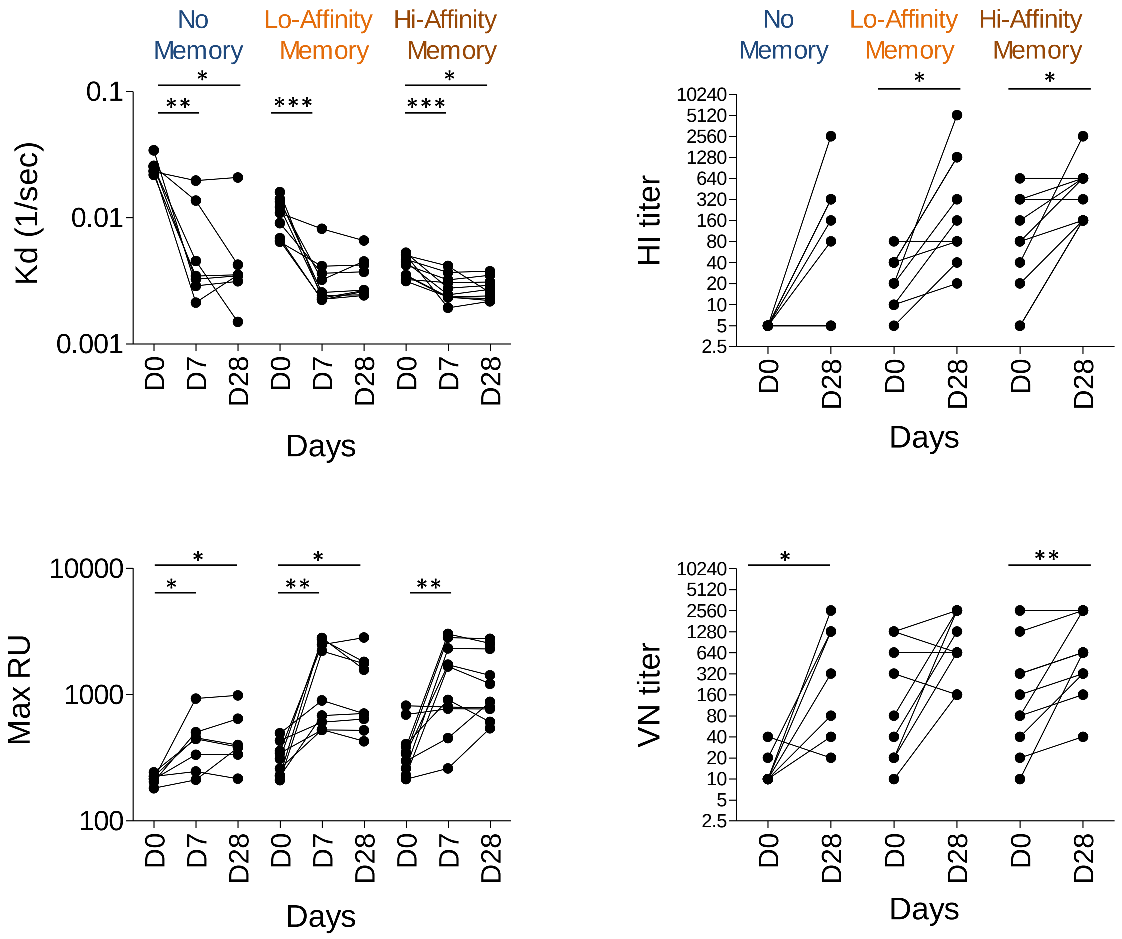


The amount (Max RU) and the avidity (Kd) of serum polyclonal IgG specific for pH1N1 HA1 at day 0, 7, and 28 post-TIV in the three subgroups (as indicated in Fig. 2b) were determined by surface plasmon resonance. The HI and VN titers against pH1N1 were determined at day 0 and 28. Paired t-test. p-value * <0.05, ** <0.01, ***< 0.001.

**Figure S2: The increase of ICOS$^{+}$ CXCR3$^{+}$ cTfh cells in absolute numbers correlates with the increase in Ab avidity**

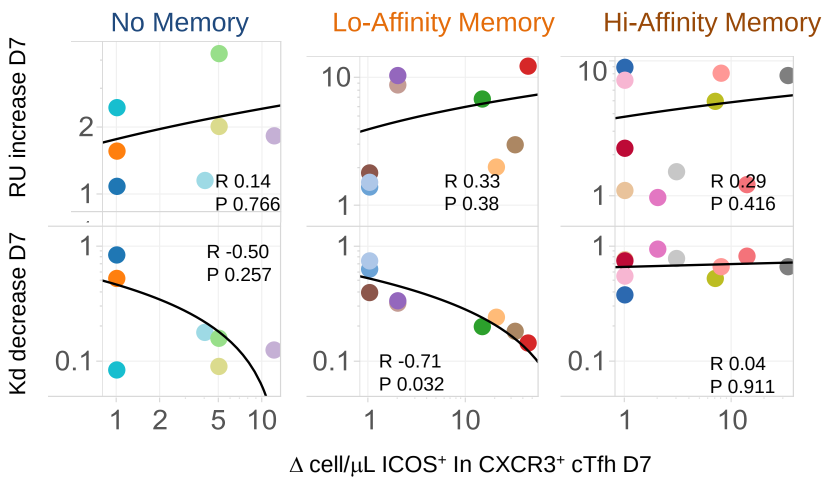


The correlation between the increase of ICOS$^{+}$PD-1$^{+}$CXCR3$^{+}$ cTfh cells in absolute number at day 7 post-TIV and the fold increase of Max RU/the fold decrease of Kd at day 7 in each group. Spearman R and p-value are indicated.

**Figure S3: Classification of the subjects according to the baseline titers**

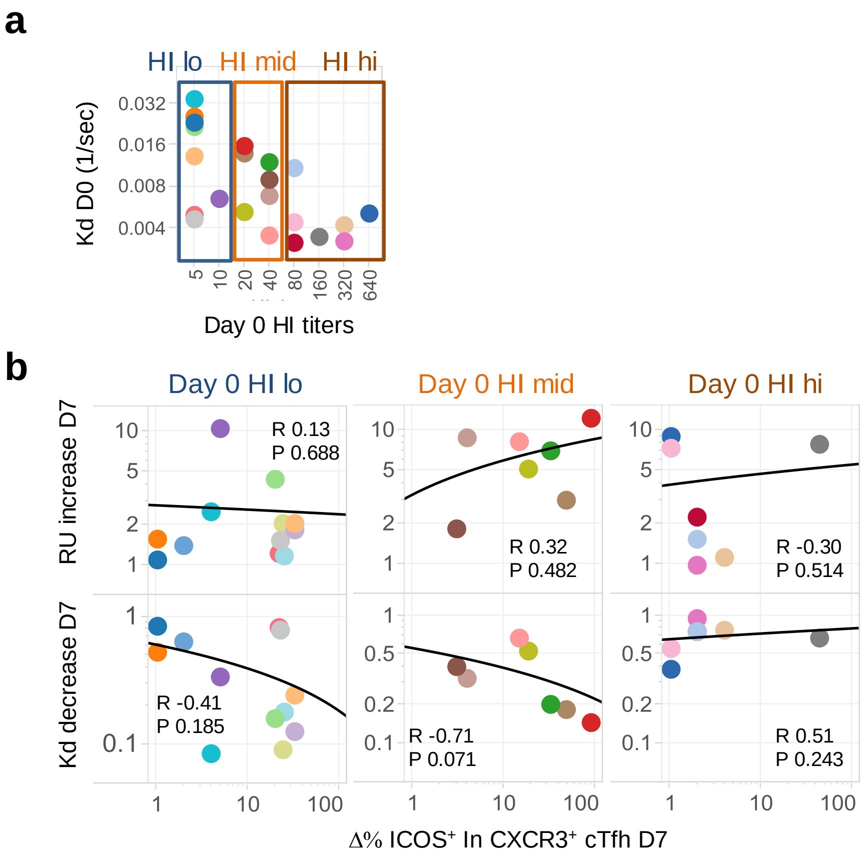


**a.** Classification of the subjects according to the baseline titers (HI low ≤ 10, n=12; HI mid between 20 and 40, n=7; HI high ≥ 80, n=7).

**b.** The correlation between the increase of ICOS$^+$PD-1$^+$CXCR3$^+$ cTfh cells at day 7 post-TIV and the fold increase of Max RU/the fold decrease of Kd at day 7 in each group.

**Figure S4: cTfh cells at day 7 post-TIV contain cells specific for HA and M1**

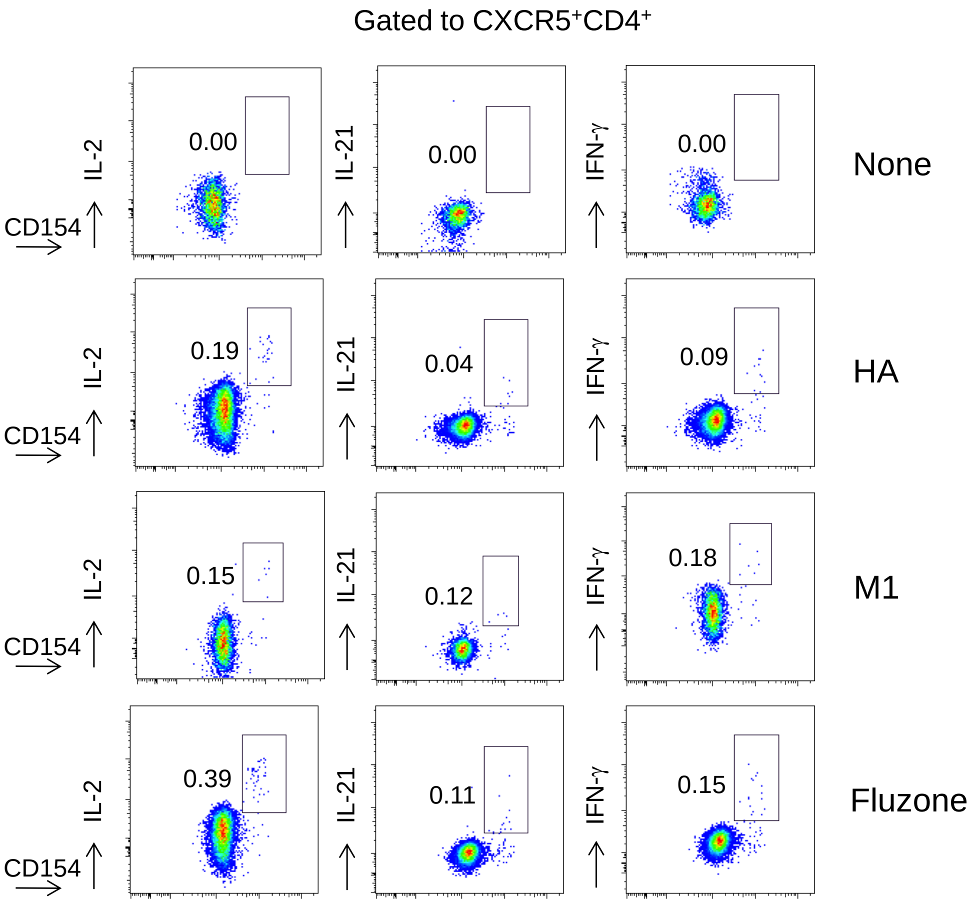


The CD154 assay was performed with PBMCs at day 7 post-TIV and overlapping peptide libraries derived from the proteins of the 2009 pandemic H1N1 strain. The results with overlapping peptides of hemagglutinin (HA) and matrix protein 1 (M1) in one donor are shown. The frequency of CD154$^{+}$cytokine$^{+}$ cells within CXCR5$^{+}$CD4$^{+}$ T cells is indicated. The result with the peptide diluent alone (none) is provided as negative control. Of note, the stimulation with Fluzone also induced CD154$^{+}$cytokine$^{+}$ cells within CXCR5$^{+}$CD4$^{+}$ T cells.

**Figure S5: CXCR5$^-$ CD4$^+$ T cells at day 7 post-TIV contain cells specific for HA and M1**

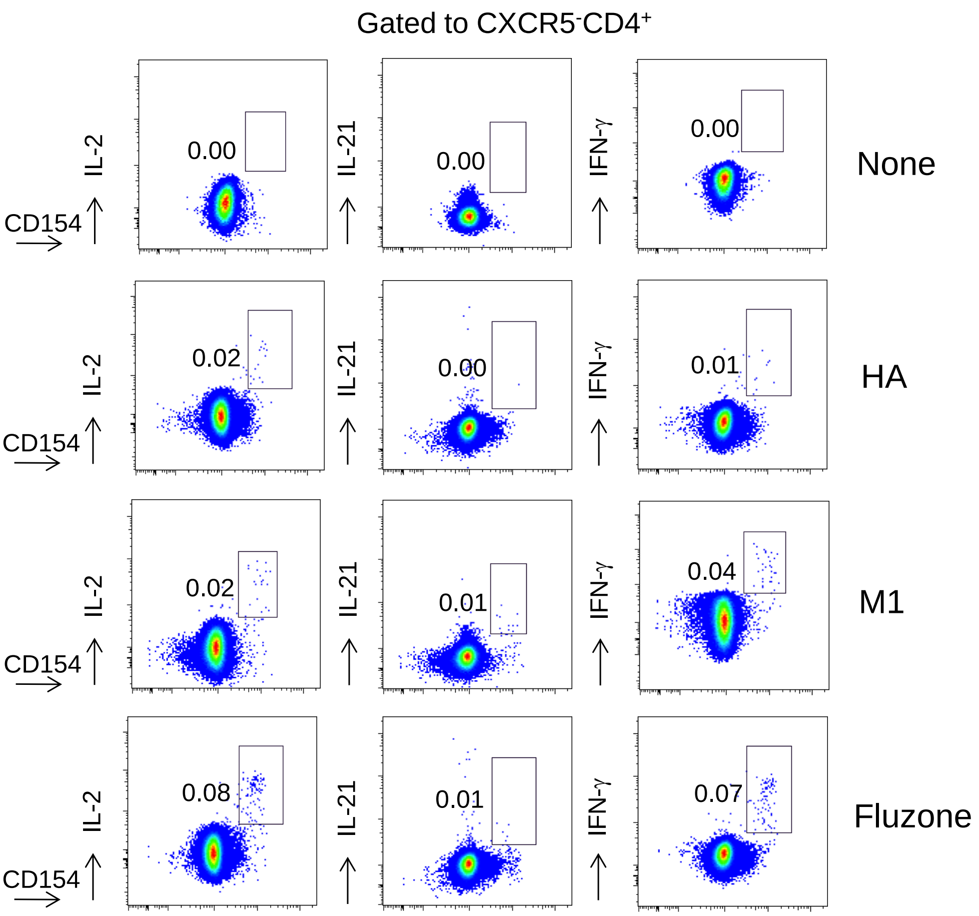


The frequency of CD154$^+$cytokine$^+$ cells within CXCR5$^-$CD4$^+$ T cells in the same experiments shown in Fig. S4 is shown.
